# Cognitive Functioning in Patients with Bipolar Disorder: Association with Depressive Symptoms and Alcohol Use

**DOI:** 10.1371/journal.pone.0013032

**Published:** 2010-09-28

**Authors:** Marieke J. van der Werf-Eldering, Huibert Burger, Esther A. E. Holthausen, André Aleman, Willem A. Nolen

**Affiliations:** 1 Department of Psychiatry, University Medical Centre Groningen, University of Groningen, Groningen, The Netherlands; 2 Department of Epidemiology, University Medical Centre Groningen, University of Groningen, Groningen, The Netherlands; 3 BCN Neuroimaging Centre, University of Groningen, Groningen, The Netherlands; James Cook University, Australia

## Abstract

**Background:**

Cognitive dysfunction is clearly recognized in bipolar patients, but the degree of impairment varies due to methodological factors as well as heterogeneity in patient populations. The goal of this study was to evaluate cognitive functioning in bipolar patients and to assess its association with depressive symptoms. Post hoc the relationship with lifetime alcohol use disorder was explored.

**Methodology/Principal Findings:**

The study included 110 bipolar patients and 75 healthy controls. Patients with severe depressive symptoms, (hypo)manic symptoms and current severe alcohol use disorder were excluded. Diagnoses were evaluated via the Mini-International Neuropsychiatric Interview. Cognitive functioning was measured in domains of psychomotor speed, speed of information processing, attentional switching, verbal memory, visual memory, executive functioning and an overall mean score. Severity of depression was assessed by the Inventory of Depressive Symptomatology-self rating. Patients were euthymic (n = 46) or with current mild (n = 38) or moderate (n = 26) depressive symptoms. Cognitive impairment was found in 26% (z-score 2 or more above reference control group for at least one domain) of patients, most prominent in executive functioning (effect size; ES 0.49) and speed of information processing (ES 0.47). Depressive symptoms were associated with dysfunction in psychomotor speed (adjusted beta 0.43; R^2^ 7%), speed of information processing (adjusted beta 0.36; R^2^ 20%), attentional switching (adjusted beta 0.24; R^2^ 16%) and the mean score (adjusted beta 0.23; R^2^ 24%), but not with verbal and visual memory and executive functioning. Depressive symptoms explained 24% of the variance in the mean z-score of all 6 cognitive domains. Comorbid lifetime alcohol use (n = 21) was not associated with cognitive dysfunction.

**Conclusions/Significance:**

Cognitive dysfunction in bipolar disorder is more severe in patients with depressive symptoms, especially regarding speed and attention. Therefore, interpretation of cognitive functioning in patients with depressive symptoms should be cautious. No association was found between cognitive functioning and lifetime comorbid alcohol use disorder.

## Introduction

During mood episodes [1]–[3], as well in euthymic phases [4], [5] bipolar patients show cognitive impairment in several neuropsychological domains. The degree of cognitive impairment varies extensively across studies due to methodological factors as well as the heterogeneity of illnesses and patient characteristics [3], [4], [6]–[11], as commonly seen in daily clinical practice. Sometimes clinicians may request for a neuropsychological assessment, since well-known social and occupational problems in bipolar patients [12]–[14] partly seem to be due to cognitive impairment [15]–[21]. An important, yet unanswered, question is how to interpret the test results in the presence of mood symptoms or long standing alcohol use. Research looking for putative cognitive endophenotypes [6], [8], [22] explicitly rules out patients characterized by commonly seen illness characteristics, and thereby limits the generalizability of these study results; after all bipolar patients are known to be euthymic for not more than 50% of time [23]–[25] and many suffer from comorbid substance use disorders, mostly alcohol misuse [26], [27]. Also, no consensus is reached about the most appropriate cognitive test battery that should be used or about the most appropriate threshold value delineating impaired from unimpaired cognitive functioning. Prior research in bipolar patients mainly reported cognitive functioning in terms of group means. An alternative approach, possibly more applicable for clinical practice, is the use of cut-off scores, highlighting the heterogeneity within the patient samples. Although arbitrary, a test result exceeding the mean of the reference group with more than 2 standard deviations is commonly considered to indicate impaired cognition [21], [28], [29].

In the current study cognitive functioning in bipolar patients as seen in daily clinical practice was evaluated. This was accomplished by including a relatively unselected group of bipolar outpatients. The extent and kind of cognitive impairment compared to healthy controls was assessed using an extensive cognitive battery. Furthermore, the association of cognitive functioning with severity of depressive symptoms was explored. In a post hoc analysis we also explored the association of cognitive functioning with lifetime alcohol use disorder. Results are expressed in group means, as well as proportions cognitively impaired.

## Methods

### Ethics Statement

This study was conducted according to the principles expressed in the Declaration of Helsinki. The study was approved by the Institutional Review Board of the University Medical Centre Groningen (reference numbers METc2005.236 and METc2007.200). All participants provided written informed consent for the collection of data and subsequent analysis.

### Participants

Recruitment of bipolar patients and healthy controls (age 18–65 years) took place between October 2005 and December 2008. General exclusion criteria were: mental retardation (IQ<70) or a known systemic or neurological disease which could influence cognitive functioning. Bipolar patients had to meet DSM-IV criteria for bipolar I, II or not otherwise specified disorder, confirmed by the Mini-International Neuropsychiatric Interview (MINI) [30]. Mild to moderate depressive symptoms were allowed, defined as a score of ≤38 points [31], [32] on the 30 item-Inventory of Depressive Symptomatology-self rating (IDS-SR) [33]. Patients with (hypo)manic symptoms, defined as >7 points on the Young Mania Rating Scale (YMRS) [34] were excluded. Regarding alcohol use disorders, patients were only excluded when they currently needed treatment in a specialized setting. Controls were systematically interviewed to exclude participants with any current or lifetime major psychiatric disorder, which included alcohol and substance use disorder. In addition, controls were excluded in case of a positive first degree family history for these disorders.

After screening for inclusion and exclusion criteria, 191 out of 261 patients from the outpatient clinic for bipolar disorder of the University Medical Centre Groningen were found eligible. No informed consent was obtained from 71 patients, for the following reasons: too busy (n = 22), fear for instability due to tests (n = 6) or other unspecified reasons (n = 43). Non-participants did not differ from the final bipolar sample in age (t = 1.32, p = 0.19), education level (t = 1.48, p = 0.14), gender (χ^2^ = 0.37, p = 0.54) or subtype of bipolar disorder (χ^2^ = 1.15, p = 0.56). A total of 120 bipolar patients were tested, but due to missing data (n = 4) and an IDS-SR score above 38 (n = 6), the final sample consisted of 110 participants. A total of 75 healthy controls were recruited using flyers in the university and hospital and by advertisements in a local newspaper. Healthy controls received 15 Euros (approximately 19 US Dollars) after participation in this study.

### Clinical Evaluation

All assessments and tests were uniformly performed by trained psychologists. Lifetime and current attention deficit and hyperactivity disorder, lifetime and current alcohol and other substance use disorders, as well as current psychotic features were assessed using the MINI. Illness characteristics were provided by the clinician via the Questionnaire for Bipolar Disorder (QBP; an adaption of the Enrolment Questionnaire as previously used in the Stanley Foundation Bipolar Network) [35], [36]. In case of mismatch between MINI and QBP results, diagnoses were checked with the treating clinician. Level of education was based on the Dutch educational system which differentiates already after primary school into different levels, ranging from 1: primary school up to 6: PhD or higher degree obtained.

### Neurocognitive Assessment

The composition of the cognitive test battery was based on existing literature and experience with the target group in clinical practice. The battery included seven cognitive domains, consisting of nine different tests, yielding 16 outcome variables.

The domain “psychomotor speed” was derived from the reaction time test of the Cambridge Neuropsychological Test Automated Battery (CANTAB) system [37]. The corresponding outcome variables in this study were the variables simple movement time (in milliseconds) and five-choice movement time (in milliseconds). For the domain “speed of information processing” the Stroop Colour and Word Test (SCWT) [38] and the reaction time test of the CANTAB system [37] were used for the outcome variables Stroop time 1 (words; in seconds), Stroop time 2 (colours; in seconds), simple reaction time (in milliseconds) and five-choice reaction time (in milliseconds). For the domain “attentional switching” the Continuous Performance Task, based on research of Smid et al. [39] was used. After a one minute practice session, two 5 minutes task-blocks (either CPT-Q or CPT-HQ condition) were performed, in which 15% of the stimuli were target stimuli demanding a response. A reliable score of attentional switching was used as outcome variable, composed of the difference score of hits in CPT-Q version minus hits in CPT-HQ version. The domain “verbal memory” was derived from the California Verbal Learning Test [40] for the outcome variables CVLT-trial 1 to 5 (verbal learning) and CVLT-number of words long term free recall. The Pattern Recognition Memory (PRM) test of the CANTAB system [37] was used to create the domain “visual memory” by calculating the outcome variables PRM-immediate correct numbers and PRM-delayed correct numbers. The domain “cognitive flexibility/planning” was derived from the Zoo map task as subtest from Behavioural Assessment of the Dysexecutive Syndrome (BADS) [41] and Stockings of Cambridge (SOC) test from the CANTAB system [37]. The outcome variables were the sum score of the raw scores of part 1 and part 2 from the Zoo map task, as well as the number of problems solved in minimal moves from the SOC. For the domain “executive functioning/working memory” the Spatial Working Memory (SWM) test of the CANTAB system [37] and the SCWT [38] were chosen to calculate the outcome variables of number of SWM-between errors for 8-box problems, SWM Strategy (counting number of times the subject begins a new search with the same box) and interference score of the SCWT (seconds).

In addition, premorbid intelligence (IQ) was estimated with the National Adult Reading Test (NART) [42]. Detailed descriptions of the pen-and-paper measures are provided by Lezak et al. [43]. Robbins et al. [37] discussed the CANTAB tests. The total cognitive test battery was administered within about 2½ hours, with one break if necessary.

### Statistical Analyses

Differences between bipolar patients and healthy controls on demographic variables were examined by means of independent t-tests for continuous variables or chi-square tests for categorical variables. All cognitive variables were assessed for normal distribution. When variables in the controls were normally distributed, the scores of controls and patients were transformed into z-scores using the mean and standard deviations of the control group. Otherwise, test scores were transformed to approximate normality by quadratic or log transformation. Higher scores indicated poorer cognition. To correct for age difference between patients and controls age-adjusted z-scores for the patients were calculated using linear regression. Differences in age-corrected z-scores between patients and controls were tested using unpaired t-tests. The analyses went on with those cognitive tests that showed statistically significant differences between patients and controls. Cognitive domain scores were created by calculating the mean z-value of the pertaining cognitive variables. In addition, a mean score was created by averaging the z-scores of all six domains. For each cognitive domain the effect size (ES) was calculated as the difference between the mean age-corrected z-scores of controls and bipolar patients. This measure of effect size (Glass's Δ) can be interpreted as a modified Cohen's d, in the sense that it is based on control group data only [44]. ES were calculated for all cognitive domains and, if necessary, additionally corrected for the potential confounders gender, educational level and premorbid IQ using multiple linear regression analyses.

To study the association of depressive symptoms with cognitive functioning we performed linear regression analyses with the age-corrected z-scores for cognition as the dependent variable, and the IDS-SR total score and potential confounders gender, education and IQ as the independent variables. The continuous IDS-SR total score was divided by 13 and consequently the beta's reported are per 13 points on the IDS-SR. The choice of 13 points is essentially arbitrary but approximately corresponds to shifting from the level of none (0–13) to mild (14–25) or from mild to moderate (25–38) depressive symptoms [31], [32]. We supplied each beta with the R^2^ (explained variance) as an indicator of model fit. In addition to the analysis of depressive symptoms as a continuum we analyzed depressive symptoms categorized as euthymic, mild, or moderate depressive symptoms. The same approach was followed for lifetime alcohol use disorder with the understanding that this variable was dichotomous only (present/absent). The linear regression model assumptions of normality, linearity and homoscedasticity were assessed using residual plots.

In addition to analyses of cognitive function in terms of group means, we analysed the proportions of cognitive impairment. To this end cognitive impairment in a patient was defined as a z-score of 2 or more above the reference control group for at least one domain; this comes down to a 2.5% prevalence of cognitive impairment per domain in the reference control group. As the analyses of continuous scores as described above are associated with optimal statistical power we refrained from additionally testing these categorical data. Statistical significance was defined as p<0.05, two sided, except for the reduction of the cognitive battery in which the statistical significance was defined as p<0.25; the use of this more liberal significance level is advocated during screening of variables for inclusion in subsequent analyses using univariable analyses [45]. All analyses were performed using Statistical Package for the Social Sciences (SPSS) Version 16.0 [46].

## Results

### Sample Characteristics

Demographical and clinical characteristics of bipolar patients and healthy controls were listed in Table 1. The study included 91 bipolar I patients (82.7%) and 19 bipolar II patients (17.3%). Patients were on average 5 years older than controls (t = −2.52, p = 0.01). Patients were euthymic (n = 46; IDS-SR score <14) or known with mild (n = 38; IDS-SR score 14–25) and moderate (n = 26; IDS-SR score 26–38) [31], [32] depressive symptoms. More than half of the patients never experienced psychotic features. Only 3 patients (2.7%) were medication-free; most other patients used 1 (52.7%, n = 58) or 2 (30.9%, n = 34) different psychotropic drugs, mostly lithium (61.8%, n = 68) and anticonvulsants (43.6%, n = 48), all in therapeutic dosages or with therapeutic plasma levels. Lifetime alcohol use disorder was present in 19.1% (n = 21) of cases. Thirteen patients from this group were also known with a current alcohol use disorder.

**Table 1:** Characteristics of Participants.

	Patients	Controls	Test £	p
	N = 110	N = 75		
Age (yrs), mean (SD)	45.7 (10.7)	40.8 (14.4)	−2.52	0.01 *
Female gender, n (%)	67 (60.9)	48 (64.0)	0.18	0.67
Premorbid IQ, mean (SD)	106.5 (9.2)	106.6 (9.9)	0.08	0.94
Education level (1–6), mean (SD)	3.6 (1.0)	3.7 (1.1)	0.53	0.60
Duration of illness (yrs), mean (SD)	20.8 (12.6)	-		
IDS-SR, mean (SD)	17.3 (10.0)	-		
YMRS, mean (SD)	0.5 (1.3)	-		
Lifetime psychotic features, n (%)	51 (46.4)	-		
Comorbidity, n (%)				
Lifetime ADHD	1 (0.9)	-		
Lifetime alcohol use disorder	21 (19.1)	-		
Current alcohol use disorder	13 (11.8)	-		
Lifetime other substance use	6 (5.5)	-		
Current other substance use	0 (0)	-		
Type of medication, n (%) $		-		
Lithium	68 (61.8)	-		
Anticonvulsants ‡	48 (43.6)	-		
Antipsychotics	27 (24.5)	-		
Antidepressants	19 (17.3)	-		
Benzodiazepines	8 (7.3)	-		

*p<0.05

$ 3 patients were medication-free

‡ 3 patients used 2 types of anticonvulsants

£ χ^2^ tests were used for categorical data and the unpaired t-test was used for continuous data

### Selection of the Cognitive Test Battery

Two cognitive outcome variables were eliminated from the battery, since they did not discriminate between healthy controls and bipolar patients at an alpha of 0.25, namely the sum score of raw score part 1 and part 2 from the Zoo map task (t = −2.47, p = 0.49) and the number of problems solved in minimal moves from the SOC (t = −0.18, p = 0.86), which together formed the whole domain of cognitive flexibility/planning (see Table 2 for raw neuropsychological test results and Table 3 for age-adjusted z-scores). Thus, further results refer to the remaining 14 cognitive outcome variables, covering six domains.

**Table 2:** Raw Data of the Neuropsychological Tests from 110 Bipolar Patients and 75 Healthy Controls.

Cognitive variables	Patients		Controls	
	Mean	sd	Mean	sd
Simple movement time (msec)	462.88	150.45	416.19	109.20
Five-choice movement time (msec)	428.13	134.33	385.30	101.33
Simple reaction time (msec)	367.47	111.08	327.67	72.77
Five-choice reaction time (msec)	386.97	97.06	344.83	57.66
Stroop time 1 (word; sec)	45.78	9.74	41.79	7.07
Stroop time 2 (colour; sec)	59.45	13.48	54.61	7.99
Difference CPT hitrate version Q minus HQ	0.05	0.07	0.03	0.07
CPT hitrate version Q (% correct)	0.99	0.03	0.99	0.06
CPT hitrate version HQ (% correct)	0.94	0.08	0.97	0.06
CVLT - verbal learning (total nr correct resp)	51.92	12.28	57.24	8.01
CVLT – long term free recall (nr correct resp)	11.65	3.36	13.15	2.31
PRM – number correct immediate	10.53	1.66	11.01	1.48
PRM – number correct delayed	9.20	2.05	10.04	1.71
SOC – problems solved in minimal moves (nr correct)	8.29	1.94	8.36	1.98
Zoo map task (total score) of BADS	12.99	3.80	13.72	3.29
SWM –between errors 8 boxes (nr correct resp)	22.38	12.75	14.69	12.04
SWM – strategy (efficiency score)	34.21	6.21	31.25	6.55
Stroop interference (difference rate; sec)	7.36	24.80	−0.11	10.16

CPT: Continuous Performance Task; CVLT: Dutch version of California Verbal Learning Test; PRM: Pattern Recognition Memory; SWM: Spatial Working Memory; SOC: Stockings of Cambridge; BADS: Behavioural Assessment of the Dysexecutive Syndrome; msec: milliseconds; nr: number; resp: responses; sd: standard deviation; sec: seconds.

**Table 3:** Age-adjusted Cognitive Z-scores of 110 Bipolar Patients.

Domains and pertaining variables	Mean	SD	t test	p
Psychomotor speed				
Simple movement time †	0.33	1.3	−1.84	0.07 *
Five-choice movement time †	0.32	1.3	−1.88	0.06 *
Speed of information processing				
Simple reaction time †	0.43	1.5	−2.41	0.02 *
Five-choice reaction time †	0.55	1.6	−3.06	0.00 *
Stroop time 1 (word) †	0.41	1.3	−2.53	0.01 *
Stroop time 2 (colour) †	0.50	1.7	−2.60	0.01 *
Attentional switching				
Difference CPT hitrate version Q minus HQ †	0.30	1.0	−2.06	0.04 *
Verbal memory				
CVLT - verbal learning (total of trial 1–5) †	0.50	1.5	−2.89	0.00 *
CVLT – long term free recall	0.34	1.0	−2.19	0.03 *
Visual memory				
PRM – number correct immediate	0.33	1.0	−1.95	0.05 *
PRM – number correct delayed	0.41	1.0	−2.71	0.01 *
Cognitive flexibility/planning				
SOC – problems solved in minimal moves †	−0.11	0.9	0.69	0.49
Zoo map task (total score) of BADS	0.04	1.1	−0.14	0.89
Executive functioning/working memory				
SWM –between errors 8 boxes	0.43	0.8	−3.49	0.00 *
SWM – strategy †	0.30	0.8	−2.32	0.02 *
Stroop interference †	0.73	2.4	−2.83	0.01 *

Healthy controls (n = 75) were used as reference score.

For all cognitive measures: higher values indicate worse performance.

*p<0.25.

† normally distributed

CPT: Continuous Performance Task; CVLT: Dutch version of California Verbal Learning Test; PRM: Pattern Recognition Memory; SWM: Spatial Working Memory; SOC: Stockings of Cambridge; BADS: Behavioural Assessment of the Dysexecutive Syndrome.

### Extent and Kind of Cognitive Dysfunctions

**Figure 1:**
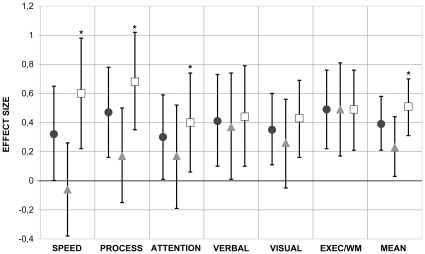
Cognitive Performance in the Total Group and Subgroups of Bipolar Patients. Total group (n = 110; dots), the euthymic subgroup (n = 46; triangle) and depressed subgroup (n = 64; square), with healthy controls (n = 75) used as reference score. Values are effect sizes, corrected for age. Error bars are 95% confidence intervals (95%CI). Statistical significance for group differences between bipolar patients and healthy controls was defined as p<0.05, shown in the figure as 95%CI which does not cross the base-line. Statistical significance (p<0.05) of sub-group differences were marked with an asterix (*) and were based on continuous depression scores. Speed  =  psychomotor speed; Process  =  speed of information processing; Attention  =  attentional switching; Verbal  =  verbal memory; Visual  =  visual memory; Exec/WM  =  executive functioning/working memory; Mean  =  mean z-score of all 6 cognitive domains.

Mean age-adjusted domain specific z-scores of patients were significantly different from controls in the range of small effect sizes (i.e. ES<0.5), with relatively large confidence intervals (see Figure 1). In the total bipolar cohort, largest ES were found for executive functioning/working memory and speed of information processing. After further correction for gender, education and IQ, results were similar.

A total of 29 bipolar patients (26.4%) were defined as cognitively impaired. Except for visual memory, dysfunctions were present in 11.8% (n = 13) of cases in the domain verbal memory, 10.9% (n = 12) in speed of information processing, 9.1% (n = 10) in attention, 8.2% (n = 9) in executive functioning and 7.3% (n = 8) in psychomotor speed. Heterogeneity within the cognitively impaired group was also illustrated by the number of impaired domains: most frequently one (51.7%, n = 15) or two (31.0%, n = 9) domains were impaired, and only in 17.3% (n = 5) of cases in the range of 3 to 5 domains.

### Association between Depressive Symptoms and Cognitive Functioning

Data in Table 4 are beta's, adjusted for age and additionally for gender, education and IQ, since these corrections led to substantial differences. The assumptions of linear regression analysis were found to be sufficiently met. An increase of 13 points on the IDS-SR total score, approximately comparable with an increase of one level of depression severity was modestly associated with psychomotor speed, speed of information processing, attentional switching and the mean score. The proportion variance explained (R^2^) ranged from 7 to 25% for the various domains of cognitive functioning and 24% for the mean z-score of all 6 cognitive domains.

**Table 4:** Associations between Depressive Symptoms and Cognitive Performance.

	Beta †	95%CI	R^2^	p
Speed	0.43	0.12; 0.73	0.07	0.01 *
Process	0.36	0.07; 0.64	0.20	0.02 *
Attention	0.24	0.01; 0.47	0.16	0.04 *
Verbal	0.20	−0.06; 0.47	0.25	0.13
Visual	0.09	−0.11; 0.29	0.21	0.39
Exec/WM	0.04	−0.23; 0.30	0.05	0.78
Mean	0.23	0.06; 0.39	0.24	0.01 *

For all cognitive measures: Beta's are corrected for age, gender, education and IQ. Higher values indicate worse performance.

*p<0.05.

95%CI: 95% confidence interval.

† All beta's are regression coefficients indicating the mean change in cognitive performance, associated with an increase of 13 points IDS-SR score.

R^2^: explained variance.

Speed  =  psychomotor speed; Process  =  speed of information processing; Attention  =  attentional switching; Verbal  =  verbal memory; Visual  =  visual memory; Exec/WM  =  executive functioning/working memory; Mean  =  mean z-score of all 6 cognitive domains.

To further illustrate the effect of depressive symptoms on cognition, the data of the euthymic (n = 46; IDS-SR score <14) and depressed (n = 64; IDS-SR score >13) patients were added to Figure 1. Expressed as proportion impaired, 13% (n = 6) of euthymic patients, 37% (n = 14) of patients with mild depressive symptoms and 35% (n = 9) of patients with moderate depressive symptoms could be defined as cognitively impaired.

### Association between Lifetime Alcohol Use Disorder and Cognitive Functioning

Data in Table 5 are beta's, adjusted for age only, since further correction for gender, education, IQ and additionally for depressive symptoms led to similar results. The assumptions of linear regression analysis were found to be sufficiently met. Lifetime alcohol use disorder (n = 21, 19.1%) in bipolar patients was not associated with the mean value of all six domains (age-adjusted beta −0.04, 95% confidence interval: −0.39; 0.31; R^2^ 0.00), nor with any of the separate cognitive domains.

**Table 5:** Associations between Lifetime Alcohol Use Disorder and Cognitive Performance.

	Beta †	95%CI	R^2^	p
Speed	−0.36	0.95; 0.22	0.01	0.22
Process	−0.08	−0.67; 0.51	0.00	0.79
Attention	0.12	−0.34; 0.59	0.00	0.60
Verbal	0.40	−0.17; 0.96	0.02	0.17
Visual	−0.13	−0.55; 0.30	0.00	0.55
Exec/WM	−0.19	−0.70; 0.32	0.01	0.47
Mean	−0.04	−0.39; 0.31	0.00	0.82

For all cognitive measures: Beta's are corrected for age. Higher values indicate worse performance.

*p<0.05

95%CI: 95% confidence interval

† All beta's are regression coefficients indicating the change in cognitive performance, associated with the presence of lifetime alcohol use disorder.

R^2^: explained variance

Speed  =  psychomotor speed; Process  =  speed of information processing; Attention  =  attentional switching; Verbal  =  verbal memory; Visual  =  visual memory; Exec/WM  =  executive functioning/working memory; Mean  =  mean z-score of all 6 cognitive domains.

## Discussion

The results of the current study confirm a significant impairment of cognitive functioning in bipolar disorder [2]–[6], [8]. However, to our knowledge this is the first study which explicitly evaluated the effect of the severity of depressive symptoms and lifetime alcohol use disorder on different types of cognitive domains. Therefore, also patients with mild or moderate depressive symptoms were included. Compared to healthy controls (and using a statistical cut off score) around 10% of euthymic bipolar patients and more than one third of patients with mild or moderate depressive symptoms were found to be cognitively impaired.

During euthymia cognitive problems in domains of executive functioning, verbal memory [4], [5], [8], as well as attention and processing speed [4] are often present in bipolar patients. These dysfunctions seem to be more prominent within an acute phase of bipolar disorder [1]–[3], [47], [48]. This notion is supported by our results, especially for the domain of cognitive speed. In the total bipolar sample, including patients in the range from euthymic to moderate depressive symptoms, diffuse results with smaller effect sizes were found in all domains, except for the domain cognitive flexibility/planning which did not discriminate between patients and healthy controls.

Depressive symptoms were associated with dysfunctions in psychomotor speed (adjusted beta 0.43; R^2^ 7%), speed of information processing (adjusted beta 0.36; R^2^ 20%), attentional switching (adjusted beta 0.24; R^2^ 16%) and the mean score (adjusted beta 0.23; R^2^ 24%), but not with verbal and visual memory and executive functioning. Nevertheless, depressive symptoms explained a large proportion of the variance in some of the cognitive domains, indicating that the effect is substantive. To our knowledge, only one previous two-year follow up study by Frasch et al. [49] reported the effect of mood symptoms on cognitive functioning reporting effect sizes; within a depressed cohort of patients with both unipolar and bipolar depression (total n = 62), they used three experimental tasks and indirectly reported that depressive symptoms were associated with processing speed instead of verbal memory. These findings are in line with our results. Importantly, subclinical mood symptoms (e.g. Hamilton depression scores <8, comparable with IDS-SR scores <14) have been stated as serious confounders [50], but a recent meta-regression did not show any impact of such low scores on any of the reported cognitive measurements in euthymic bipolar patients [6].

Another study goal was to evaluate the possible, largely undetermined [51], [52] effect of lifetime comorbid alcohol use disorder on cognitive functioning. No (additional) effect of lifetime alcohol use disorder was found in any of the tested domains. This finding is in line with recently reported data by Sanchez-Moreno et al. [53], who compared cognitive function of 30 bipolar patients with and 35 without a lifetime history of strictly defined alcohol abuse or dependency. Other prior studies are ambiguous, with some authors suggesting an additional decline in cognitive functioning in patients with comorbid substance use disorder [54], [55], while others did not find any relation at all [56]–[59]. In the current study we focused on the effect of lifetime, instead of current alcohol use disorder [11], because cognitive dysfunctions can remain over more than 5 years in alcoholics who finally stopped using of alcohol [60], [61] and past exposures are often not taken into account [22]. We also did not demonstrate an effect of current alcohol use disorder on any of the cognitive domains. These negative findings can be explained by the post hoc condition, as a result of which we did not explicitly recruit patients with and without lifetime alcohol use disorders. This apparently resulted in relatively small numbers of patients with lifetime (19.1%, n = 21) and with current (11.8%, n = 13) alcohol use disorder. Another reason for these small numbers is that patients with current severe alcohol use disorder were excluded.

Highlighting the heterogeneity of cognitive dysfunctions revealed cognitive impairments in 26% of our patient cohort, mainly in one variable domain. This result is difficult to compare with prior research. For example, Gualtieri and Morgan [28] defined five cognitive domains (memory, psychomotor speed, information processing speed, attention and cognitive flexibility) based on different tasks than the current study and without mood ratings. In that study thirty percent of bipolar patients were considered cognitively impaired, using 2 standard deviations below mean as cut off score. Thompson et al. [62] evaluated the magnitude of effect sizes in 11 individual cognitive tasks in euthymic bipolar patients, with (arranged by the magnitude of effect sizes) 3–42% of all patients being cognitively impaired. Iverson et al. [63] calculated five domain scores based on fully computerized cognitive tests (memory, psychomotor speed, reaction time, cognitive flexibility, and complex attention), evaluating 47 outpatients with bipolar disorder derived from a sample of convenience (no formal diagnostic interviewing or symptom rating scales) and reported 47% of patients as cognitively impaired, using one or more domain scores of 2 SD below the mean. Looking at the number of impaired cognitive domains, Martino et al. [29] stated that 38% of euthymic bipolar patients were not impaired in any of the six domains (attention, verbal memory, language, psychomotor speed, executive function and facial recognition task), 40% in 1 or 2 domains and 22% in 3 to 5 domains. Altogether this short overview shows that consensus about how to test (which domains, which tests) and how to define cognitive impairment is urgently needed.

The current study has several strengths and limitations. A major strength is the generalizability of test results. The study population comprised 60% of the patients of our outpatient clinic for bipolar disorders who fulfilled inclusion criteria other than informed consent, while refusers were not found to be different form those who gave consent on major demographic and illness characteristics. Moreover, although our academic centre is specialized in the treatment of bipolar patients, the majority of patients were not referred for tertiary care or more specialized treatment indicated by the fact that many patients receive only one or two types of medication. Finally, the exclusion rate was very low; i.e. we excluded only patients who were severely depressed or (hypo)manic, patients with severe current alcohol or drug abuse and patients with a medical comorbidity known to affect cognitive functioning. Therefore, these findings are considered representative for the population of bipolar patients as seen in clinical practice. Also, since only one patient (0.9%) was diagnosed with lifetime Attention Deficit Hyperactivity Disorder (ADHD), the results are not confounded by the presence of comorbid ADHD [51], a factor often overlooked in cognitive literature. ADHD is characterized by attention and executive problems in childhood [64] and is reported in up to 16% of adult bipolar patients [65]. Importantly, psychiatric comorbidity (e.g. anxiety disorder) did not seem to aggravate the level of cognitive dysfunction in depressed young adults [56]. The cognitive test battery used in this study is another major advantage, since it covers all relevant cognitive domains with regard to bipolar disorder, but by averaging different tasks within the corresponding domains, it reduces multiple comparisons for data analysis and avoids disproportionate emphasis of single test results [66]. Also, in this manner we expanded the number of domains that have been evaluated in depressed bipolar patients [2].

Some limitations need to be considered. In absence of (hypo)manic patients, we can only make statements about the association of depressive symptoms with cognitive functioning in bipolar patients. Furthermore, this is a cross-sectional study and therefore it does not allow drawing conclusions about the causality between cognitive functioning and the two commonly seen patient characteristics (depressive symptoms and lifetime alcohol use). Although the current study sample was large compared to previous studies in this area, it could have been too small to detect significant results, especially regarding the comparison of subgroups, including the post hoc analysis of the potential effect of lifetime alcohol use disorder. Therefore, future research should formulate a specific aim to recruit sufficiently large samples of patients from the various subgroups.

From a clinical perspective (instead of aetiological origin) we choose to study a heterogeneous patient cohort. It has never been the main focus to take into account all the possible influence of medications and psychiatric comorbid conditions. Research in this area [51], [67] is still ongoing. For example, a recent meta-analysis surprisingly showed that lithium treatment has only few and minor negative effects on cognition [68]. Finally, it is tempting to define the effect sizes of cognitive domains and effect sizes of the association with patient characteristics in terms of small, moderate or even large effect sizes, but since the lack of knowledge of clinical relevance (instead of statistical significance) these magnitudes of numbers have to be interpreted with caution. Also, it could be argued that the cut off score of 2 standard deviations is relatively conservative, but proven to detect significantly impaired persons.

In conclusion, our study confirmed and extended the knowledge of cognitive dysfunction in patients with bipolar disorder. Cognitive dysfunction is more severe in patients with depressive symptoms, especially regarding the domains of speed and attention. Therefore, assessment of cognitive functioning in patients with mild to moderate depressive symptoms should be interpreted with caution. Moreover, post hoc analysis did not show any association between cognitive functioning and lifetime comorbid alcohol use disorder.
